# Plasticity of DNA Replication Initiation in Epstein-Barr Virus Episomes

**DOI:** 10.1371/journal.pbio.0020152

**Published:** 2004-06-15

**Authors:** Paolo Norio, Carl L Schildkraut

**Affiliations:** **1**Department of Cell Biology, Albert Einstein College of MedicineBronx, New YorkUnited States of America

## Abstract

In mammalian cells, the activity of the sites of initiation of DNA replication appears to be influenced epigenetically, but this regulation is not fully understood. Most studies of DNA replication have focused on the activity of individual initiation sites, making it difficult to evaluate the impact of changes in initiation activity on the replication of entire genomic loci. Here, we used single molecule analysis of replicated DNA (SMARD) to study the latent duplication of Epstein-Barr virus (EBV) episomes in human cell lines. We found that initiation sites are present throughout the EBV genome and that their utilization is not conserved in different EBV strains. In addition, SMARD shows that modifications in the utilization of multiple initiation sites occur across large genomic regions (tens of kilobases in size). These observations indicate that individual initiation sites play a limited role in determining the replication dynamics of the EBV genome. Long-range mechanisms and the genomic context appear to play much more important roles, affecting the frequency of utilization and the order of activation of multiple initiation sites. Finally, these results confirm that initiation sites are extremely redundant elements of the EBV genome. We propose that these conclusions also apply to mammalian chromosomes.

## Abstract 2

Despite overall similarities between genomes, initiation of DNA replication and speed of duplication in different parts of the genome differs amongst EBV strains

## Introduction

Biochemical studies performed in higher eukaryotes have shown that DNA replication initiates at specific sites, or within initiation zones, suggesting the involvement of particular DNA sequences called replicators (reviewed by DePamphilis 1999). In contrast, functional studies, as well as studies of DNA replication performed in early embryos of various vertebrates and invertebrates, have suggested that initiation of DNA replication can take place with limited sequence specificity (reviewed in Gilbert 2001).

The presence of specific initiation sites and of initiation zones has also been proposed to explain the latent replication of the Epstein-Barr virus (EBV) genome in human cell lines. During latent replication, the EBV genome is maintained as a circular episome (∼175 kb in size), and the host cell provides both the replication machinery and the licensing apparatus that limit the genome's duplication to once per cell cycle (reviewed in Kieff 1996; Yates 1996). Initiation site oriP was the first initiation site identified in the EBV genome. In the presence of the viral protein EBNA1, this DNA sequence confers autonomous replication to plasmids transfected into human cell lines (Yates et al. 1984). In addition, initiation of DNA replication at oriP was recently shown to be regulated by geminin, and to correlate with the binding of various cellular components of the replication complex (Orc1, Orc2, Orc3, Orc4, Orc6, Mcm2, Mcm3, and Mcm7) (Chaudhuri et al. 2001; Dhar et al. 2001; Schepers et al. 2001; Ritzi et al. 2003). These and other reports have been interpreted as evidence that oriP contains a replicator (e.g., Koons et al. 2001). However, other initiation sites have also been described (Kirchmaier and Sugden 1998), and a study performed by two-dimensional (2D) gel electrophoresis at neutral pH has suggested the presence of a large initiation zone (Little and Schildkraut 1995). In addition, reports from different laboratories have shown that various portions of the EBV genome, including oriP, can be deleted without affecting the maintenance of the episomes in replicating cells (see Discussion and references therein). Therefore, the presence of specific replicator sequences and their relationship with the sites of initiation of DNA replication also remain to be demonstrated in this system.

We recently began to study the replication of individual EBV episomes using fluorescence microscopy (Norio and Schildkraut 2001). In a previous study, we collected various images of the Raji EBV genome (Norio and Schildkraut 2001). The analysis of those molecules demonstrated that the duplication of different EBV episomes begins at different initiation sites located within the initiation zone identified by 2D gel electrophoresis. However, the number of molecules analyzed was not sufficient to infer the precise dynamics of activation of the initiation sites (i.e., to detect events having a short life or occurring infrequently during the duplication of the episomes).

In the present study, we performed an extensive analysis of the replication dynamics of the EBV genome in two human Burkitt's lymphoma cell lines (Raji and Mutu I). By utilizing a different procedure to stretch DNA molecules we were able to collect a large number of images of the EBV genome representative of different stages of duplication. This allowed us to determine how DNA replication initiates, progresses, and terminates throughout the EBV genome and to precisely measure the duplication time of specific portions of the EBV genome.

These improvements allowed us to obtain important new results as well as to extend previous observations. Here we show that initiation events are not limited to a specific portion of the EBV genome (namely the initiation zone detected by 2D gel electrophoresis), but, unexpectedly, take place throughout the EBV genome. Multiple initiation events were also detected in individual EBV episomes. Hence, if the initiation sites do correspond to replicators, the latter must necessarily be highly redundant (present at a frequency of one or more every 20 kb).

Our new results also indicate that, in these two EBV strains, both the frequency and the order of activation of the initiation sites vary considerably throughout the viral genome. This variation involves initiation sites such as oriP, the sequence of which is highly conserved in the two EBV strains (Salamon et al. 2000). Hence, the utilization of the initiation sites is largely independent of their DNA sequence, and it is affected by the genomic context (i.e., the presence/absence of initiation sites activated earlier or the presence of transcription). Finally, we noticed that the initiation sites that tend to be activated earlier during the duplication of each episome are located in clusters, each of which spans several kilobases. The locations of these clusters are different in the Raji and Mutu I strains. Therefore, the utilization of the initiation sites (particularly their order of activation) appears to be regulated at the level of genomic regions rather than at the level of individual initiation sites.

## Results

### Fluorescent Hybridization Immunostaining of Individual EBV Episomes Stretched on Microscope Slides

In order to study DNA replication, we used a procedure that we call single molecule analysis of replicated DNA (SMARD). This procedure labels the replicating DNA in a way that allows us to determine the position, the direction, and the density of the replication forks in a steady-state population of replicating molecules (in this case, EBV episomes). This in turn allows us to determine how DNA replication initiates, progresses, and terminates throughout the genomic region analyzed. SMARD presents several advantages over procedures that utilize different labeling schemes and allows us to overcome most of the limiting factors that have traditionally affected studies of replication performed on DNA fibers (see Materials and Methods).

In our procedure, an asynchronous population of cells is sequentially labeled with 5′-iodo-2′-deoxyuridine (IdU) and 5′-chloro-2′-deoxyuridine (CldU) (Norio and Schildkraut 2001). The length of each labeling period is longer than the time required to completely replicate the EBV genome (3.5–4 h; see Materials and Methods). This allows some of the replicating EBV episomes to become substituted with the halogenated nucleotides along their entire length (Norio and Schildkraut 2001). The incorporation of these nucleotide analogs is later detected by immunofluorescence of individual DNA molecules stretched on microscope slides. In these molecules, the transitions from IdU to CldU mark the positions of the replication forks at the time of the switch from the first to the second labeling period (see below). Hence, the results of this analysis are presented as a series of images of EBV episomes representative of the different stages of duplication that were present at the end of the first labeling period. In addition, the use of long labeling periods makes the data collected by SMARD suitable for quantitative analysis, allowing us to calculate the duplication time of different genomic regions.

**Figure 1:**
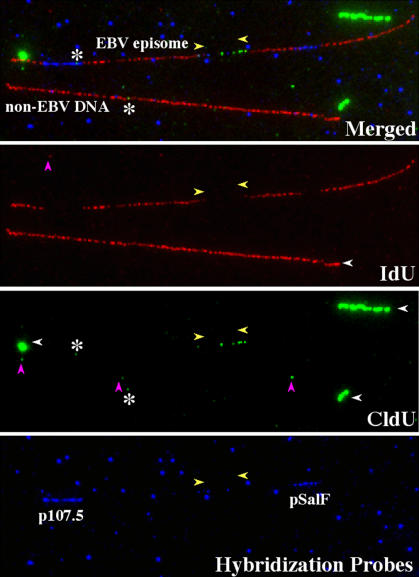
Fluorescent Hybridization Immunostaining of Individual EBV Episomes Image of two stretched DNA molecules in the same optical field. The hybridization signals (p107.5 and pSalF) and the immunostaining to detect the halogenated nucleotides are shown in different pseudocolors (red = IdU, green = CldU, and blue = hybridization probes). The top panel shows the merged image. The different color channels are shown separately in the lower panels. One of the two stretched molecules is a PacI-linearized EBV episome (molecule above) and can be recognized by the presence of the hybridization signals. The molecule below is a piece of cellular genomic DNA of similar size (no hybridization signals). The presence of the hybridization signals decreases the intensity of the immunostaining along the same portion of the EBV episome. This confirms that both halogenated nucleotides and hybridization probes are located on the same DNA molecule. The blue dots visible in the bottom panel represent hybridization background (this background was digitally removed from Figures 3B, 4B, and 6B). The EBV episome is substituted along its entire length with both IdU (red regions) and CldU (green regions). Yellow arrowheads indicate the approximate position of the replication forks at the time of the switch from the first to the second labeling period. The background visible in the red and green channels is mainly other DNA molecules containing halogenated nucleotides (white horizontal arrowheads). Some of these molecules attached to the glass before becoming fully extended and appear thick, displaying a brighter immunostaining. Small dots are also visible (magenta vertical arrowheads), sometimes overlapping with the DNA molecules (white asterisks); however, they were not considered in our analysis because they are too short to be unequivocally ascribed to DNA replication.

**Figure 2:**
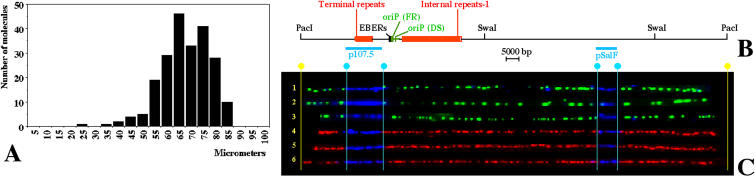
Stretching DNA Molecules by Capillary Action (A) Lengths of 219 unbroken Raji EBV episomes with a recognizable hybridization pattern. These molecules were stretched by the movement of a DNA solution between a silanized microscope slide and a nonsilanized coverslip. About 94% of these molecules have a size of 70 μm (±15 μm), corresponding to about 2.4 kb/μm. (B) Schematic of the PacI-linearized Raji EBV genome with the positions of various genetic elements shown to scale. The initiation site oriP is shown in green with the FR element and the DS element indicated by green boxes. The positions of EBER genes (black box), terminal repeats (smaller red box), internal repeats 1 (larger red box), and the restriction sites utilized in this study (PacI and SwaI) are also indicated. (C) Images of 6 PacI-linearized Raji EBV episomes aligned with the EBV map after hybridization and immunostaining of the DNA molecules and digital adjustment of length. The hybridization signals are shown in blue. Immunostaining to detect the halogenated nucleotides is shown by red and green. Vertical light blue lines indicate the positions of the ends of the hybridization probes and yellow lines, the position of the PacI site used to linearize the EBV episomes. All the molecules shown in this figure represent EBV episomes duplicated during either the first labeling period (red) or the second labeling period (green). The quality of the alignment of the images with the EBV map is comparable to the alignment previously obtained with combed EBV episomes (Norio and Schildkraut 2001). The resolution of analysis is limited to about 5 kb primarily because of discontinuities in the fluorescent signals, as previously reported for similar assays (Parra and Windle 1993; Jackson and Pombo 1998; van de Rijke et al. 2000).

In the experiments described in this study, agarose-embedded total DNA was prepared from cells labeled with halogenated nucleotides. The circular EBV episomes were converted to linear molecules by digestion with a restriction endonuclease (PacI or SwaI). After pulsed field gel electrophoresis, the EBV DNA was recovered by agarase treatment (Norio and Schildkraut 2001) and stretched on microscope slides by capillary action (see Materials and Methods). Using this procedure we obtained relatively high numbers of stretched molecules even when the starting amount of purified DNA was very small. The hybridization of specific biotinylated probes (visualized with Alexa Fluor 350-conjugated avidin; shown in blue in the figures throughout the manuscript) was used to identify the EBV molecules and their orientation (Norio and Schildkraut 2001). In addition, the halogenated nucleotides were visualized using specific monoclonal antibodies and secondary antibodies conjugated with Alexa Fluor 568 (shown in red in the figures throughout the manuscript; IdU) and Alexa Fluor 488 (shown in green in the figures throughout the manuscript; CldU). The detection procedure and the analysis of the images are described in Materials and Methods and in Figures 1 and 2. The use of long labeling periods, and the analysis of molecules substituted with the halogenated nucleotides along their entire length, present several advantages. In particular this procedure provides multiple internal controls that could not have been performed if short labeling times had been used (see Materials and Methods).

### The Raji EBV Genome Contains a Region That Usually Replicates First and a Region That Usually Replicates Last

**Figure 3:**
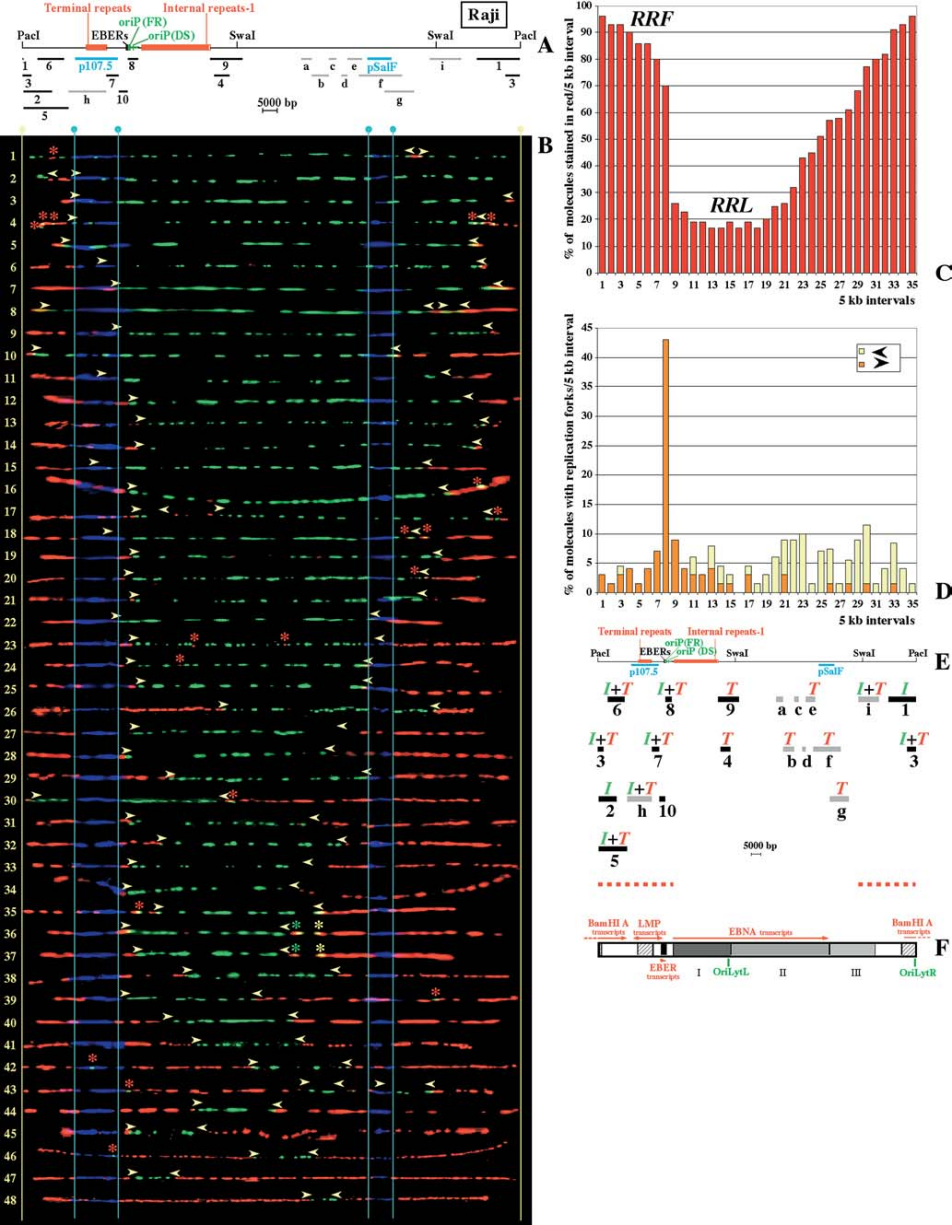
SMARD Performed on PacI-Linearized EBV Episomes Replicated in Raji Cells (A) Map of the PacI-linearized EBV genome with the positions of various genetic elements shown to scale. Below the EBV map, light blue bars indicate the positions of the hybridization probes (p107.5 and pSalF) utilized during SMARD to identify the molecules of interest and their orientation. Gray bars *(a–h),* and black bars (1–10), indicate the positions of the restriction fragments analyzed by 2D gel electrophoresis. (B) PacI-linearized Raji EBV episomes after hybridization and immunostaining of the DNA molecules (aligned with the map). These molecules incorporated both halogenated nucleotides, and the images are ordered (from 1 to 48) by increasing content of DNA labeled during the first labeling period (red). One additional molecule was unsuitable for precise measurements and is not shown. Vertical light blue lines indicate the positions of the ends of the hybridization probes and yellow lines, the position of the PacI site. Arrowheads mark the approximate position of the red-to-green transitions. Asterisks indicate the position of short colored patches not necessarily related to DNA replication. (C) Replication profile of the Raji EBV episomes. This profile was obtained using both the images shown in (B) and the images collected in a previous SMARD experiment (Norio and Schildkraut 2001), for a total of 69 episomes. Starting from the PacI site, genomic intervals of 5 kb are indicated on the horizontal axis by numbers from 1 to 35. The vertical axis indicates the percentage of molecules stained red within each 5-kb interval. (D) Profile of replication fork abundance and direction throughout the EBV genome. Genomic intervals of 5 kb are indicated on the horizontal axis as for (C). The vertical axis indicates the percentage of molecules (out of a population of 69 episomes) containing replication forks (red-to-green transitions) within each 5-kb interval. The forks moving from left to right are depicted in orange. The forks moving from right to left are depicted in yellow. (E) Map of the EBV genome aligned with the horizontal axes of histograms (C) and (D), and with the restriction fragments analyzed by 2D gel electrophoresis (black and gray bars below the map). Green *I*s indicate the presence of replication bubbles. Red *T*s indicate the presence of replication intermediates produced by random termination events. Replication bubbles were detected by 2D gel electrophoresis across the region marked by the red dashed line (approximately corresponding to the RRF). (F) Transcription of the Raji EBV genome. Red arrows mark the positions of regions that can be transcribed during latency. The level of transcription derived by nuclear run-on according to Kirchner et al. (1991) is shown as gray scale (black = highest level; white = lowest level or not transcribed). The EBER genes represent the most intensively transcribed portion of the EBV genome. Intermediate levels of transcription were detected across and downstream from the long transcription unit of the EBNA genes. According to Sample and Kieff (1990), the level of transcription along the EBNA genes region decreases from left to right (I–III). Intermediate levels of transcription were also reported for the two hatched regions. However, these regions contain either repeated sequences (the terminal repeats) or cross-hybridize with other transcribed regions (oriLytR and oriLytL); therefore, their actual level of transcription could be lower.

In order to define precisely how the Raji EBV genome replicates, we recovered the images of 245 PacI-linearized EBV episomes that incorporated halogenated nucleotides along their entire length (112 fully stained in red, 84 fully stained in green, and 49 stained in both red and green). The results of this experiment are shown in Figure 3. In the episomes that incorporated both kinds of halogenated nucleotides, the red to green transitions (arrows in Figure 3B) define the position of the replication forks at the time of the switch from the first to the second labeling period. The red portions of these molecules are nested around the ends of the PacI-linearized episomes (Figure 3B). This indicates that DNA replication proceeded in a similar manner in most of episomes.

However, the progression of DNA replication throughout the EBV genome is better described by the replication profile of the molecules analyzed (Figure 3C). This profile was obtained by dividing the map of the episomes into intervals of 5 kb (horizontal axis) and then indicating the percentage of molecules stained in red within each interval (vertical axis). From this profile we can easily identify a genomic region that usually replicates first (RRF; more frequently stained in red), a genomic region that usually replicates last (RRL; less frequently stained in red), and two transition regions.

The RRF contains the initiation sites most frequently utilized to begin the duplication of the Raji EBV episomes. More than 80% of the molecules analyzed were stained in red throughout intervals 1–7 and 31–35 (horizontal axis; Figure 3C). In the molecules representing early stages of episomal duplication (upper portion of Figure 3B), initiation events took place either within the RRF (molecules 2–21) or in adjacent portions of the EBV genome (i.e., molecule 1). Interestingly, low levels of replication bubbles had been previously detected by 2D gel electrophoresis within various restriction fragments located in the same portions of the EBV genome (black bars 1–3 and 5–7 in Figure 3A). Therefore, the initiation sites activated earlier during the duplication of each episome are located within what appears to be an initiation zone that spans several tens of kilobases (encompassing intervals 1–7 and 27–35 in Figure 3C).

The RRL appears in the replication profile of the Raji EBV episomes as a large valley (Figure 3C). The bottom of the valley spans about 40 kb (intervals 11–18), and its flat appearance indicates that throughout this region the episomes terminate their duplication with similar probability. Note, however, that termination events can also occur within the transition regions (green in molecules 43 and 48; Figure 3B). Interestingly, the level of transcription across the RRL is higher than in the rest of the Raji EBV genome, while across the RRF it is either very low or absent (Figure 3F) (Sample and Kieff 1990; Kirchner et al. 1991).

**Figure 4:**
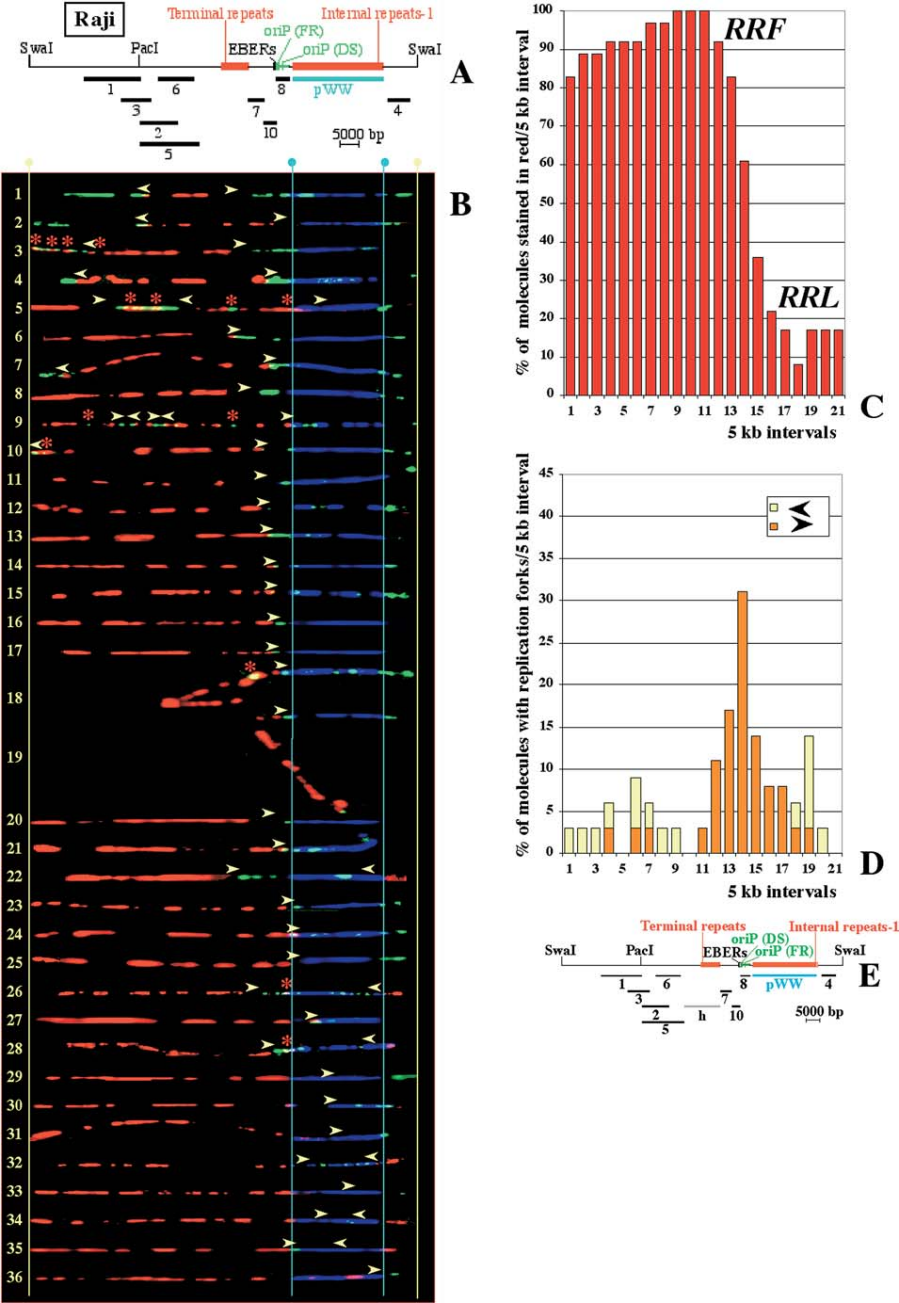
SMARD Performed on SwaI-Digested EBV Episomes Replicated in Raji Cells (A) Map of the approximately 105-kb fragment obtained by digesting EBV episomes with the restriction endonuclease SwaI. (B) Images of 36 EBV molecules ordered and marked as in Figure 3B. Molecules 18 and 19 are distorted, but the positions of the red-to-green transitions are clear. (C) Replication profile of the SwaI-digested EBV episomes shown in (B). Starting from the SwaI site, intervals of 5 kb are indicated on the horizontal axis by numbers from 1 to 21. The vertical axis indicates the percentage of molecules stained red within each 5-kb interval. (D) Profile of replication fork abundance and direction. Intervals of 5 kb are indicated on the horizontal axis as for (C). The vertical axis indicates the percentage of molecules containing replication forks in each 5-kb interval. The partitioning of the EBV genome is different from Figure 3D. Hence, the four pausing sites that in Figure 3D were located within interval 8 are here located between interval 13 and interval 14. As a consequence, the peak visible in Figure 3D is here split into two smaller adjacent peaks. (E) Map of the approximately 105-kb SwaI fragment aligned with the horizontal axes of histograms (C) and (D).

The presence of RRF and RRL was confirmed by a second SMARD experiment in which we digested the EBV episomes with SwaI. This enzyme cleaves twice in the viral genome, producing fragments of 105 and 70 kb. The larger fragment was expected to contain most of the RRF (now located near the center of DNA molecules), and a small portion of the RRL. We recovered 209 fully substituted 105-kb fragments (94 red, 79 green, and 36 red and green). These molecules were analyzed as described for the PacI-linearized EBV episomes (Figure 4). We found that both the RRF (intervals 1–13), and the RRL (intervals 17–21) encompass the same genomic sequences occupied in the previous SMARD experiment (compare Figures 3C and 4C). Initiation events located within the RRF are visible in molecules 1–4 (Figure 4B). We conclude that the results obtained by SMARD are reproducible and do not depend on the particular restriction enzyme used for digesting the DNA molecules.

### Replication Forks Move Without Significant Pausing throughout the Raji EBV Genome with the Exception of the Genomic Region near oriP

The movement of the replication forks throughout the EBV genome is described by the profiles of replication fork abundance (see Figures 3D and 4D). These profiles were obtained by dividing the map of the EBV genome into intervals of 5 kb (horizontal axis) and then indicating the percentage of molecules containing red-to-green transitions within each interval (vertical axis). As seen earlier, these transitions indicate the position, and the direction, of the replication forks at the time of the switch from the first to the second labeling period. The significant accumulation of red-to-green transitions visible within interval 8 (Figure 3D) indicates that replication forks were not moving freely across this portion of the EBV genome. A similar accumulation is visible for the SwaI-digested molecules in the same portion of the EBV genome (Figure 4D). This result was expected since four different pausing sites had been previously described within and near oriP (Little and Schildkraut 1995; Norio and Schildkraut 2001). However, no other major accumulation of forks is visible. Therefore, replication forks move mostly unimpeded across the Raji EBV genome.

From the profiles of replication fork abundance we can also determine the prevalent direction of the replication forks throughout specific portions of the EBV genome. For example, throughout most of the RRL, replication forks move in both directions at similar frequencies (yellow and orange bars within intervals 11, 13–15, and 17 in Figure 3D and within intervals 18 and 19 in Figure 4D). The bidirectional movement of the replication forks also characterizes the central portion of the RRF (intervals 4–7; Figure 4D). However, this was not evident from the profile of replication fork abundance of the PacI-linearized EBV episomes (Figure 3D) because the extremities of the molecules can be distorted or not fully stretched (such as in molecule 5 in Figure 3). As a consequence, in the PacI experiment, the position of the replication forks could not always be observed properly within the RRF. From this we conclude that within the central portions of RRF and RRL, replication forks move in both directions with a similar probability.

Within the rest of the EBV genome, however, the movement of the replication forks is mostly unidirectional (Figures 3D and 4D). For example, replication forks move mainly rightward from interval 11 throughout oriP and beyond (Figure 4D; forks infrequently moving in the opposite direction may not appear in this kind of histogram unless extremely large numbers of molecules are imaged). This direction bias is compatible with a previous 2D gel analysis of the oriP region in Raji cells (Little and Schildkraut 1995) and is not affected by the pausing of the replication fork. We conclude that the direction of movement of the replication forks is mainly a consequence of the dynamics of initiation of DNA replication throughout the viral genome.

### Active Initiation Sites Are Not Limited to the RRF

Early studies performed by electron microscopy identified Raji EBV episomes with multiple replication bubbles but could not identify the position of these initiation events (Gussander and Adams 1984). In order to detect the presence of these events and to determine their location we analyzed the immunostaining patterns of the DNA molecules described above. Multiple initiation events should produce molecules containing multiple red patches, each surrounded by green. The qualitative examination of the replication patterns shown in Figure 3B revealed some of these immunostaining patterns. In molecules 17 and 43, for example, an early initiation event apparently took place within the RRF (large region stained in red). However, shorter red regions are also present, indicating the occurrence of initiation events at later times. Throughout this manuscript, when we refer to multiple initiation events, we will mean that they occur on the same DNA molecule. In addition, if the activation of the initiation sites is not synchronous (as in the molecules described above), we will refer to the initiation events used to begin the duplication of the EBV genome as primary and any subsequent initiation event as secondary. The secondary events visible in molecules 17 and 43 are both located within the long transcription unit of the Epstein Barr nuclear antigen [EBNA] genes (see Figure 3F). In particular, molecule 43 shows a secondary initiation event that occurred when the duplication of the EBV episome was almost complete (red patch near the pSalF hybridization signal). Therefore, initiation events are not limited to the RRF of the Raji episomes. Initiation events located throughout the EBV genome, as well as multiple initiation events, were also identified in a much larger fraction of Mutu I EBV episomes (see below). We conclude that the entire EBV genome constitutes a large initiation zone, although the frequency of the initiation events is reduced throughout RRL (see below).

### DNA Replication Proceeds at Different Speeds throughout Different Portions of the Raji EBV Genome

**Figure 5:**
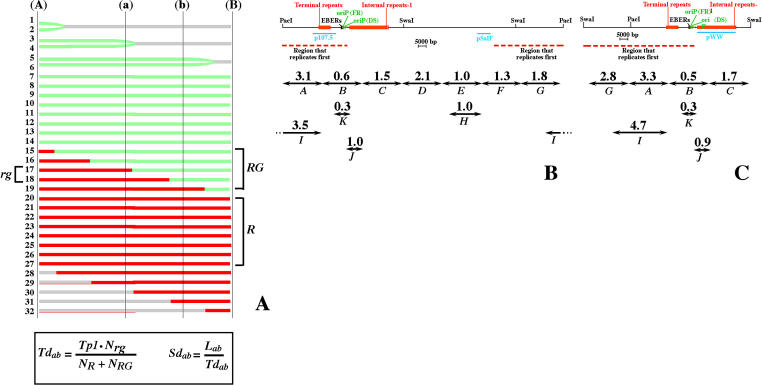
Duplication Speed of Various Segments of the Raji EBV Genome (A) A detailed procedure to calculate *Td* using SMARD was published elsewhere (Norio and Schildkraut 2001). Here we describe how to calculate *Td_ab_* for a generic genomic region (a)–(b) (i.e., any portion of the EBV genome) using information derived from the replication patterns of a longer region (A)–(B) (i.e., the whole EBV genome). Depicted are the hypothetical staining patterns for 32 DNA molecules representing the genomic region (A)–(B) after double-labeling with two halogenated nucleotides (red and green). The molecules that started and completed their duplication during the first labeling period are fully red *(R).* The molecules that started their duplication during the first labeling period, completing it during the second labeling period are stained in both red and green *(RG).* In the total population of molecules, the fraction of *R* molecules increases when the length of the first labeling period *(Tp1)* increases. The fraction of *RG* molecules is proportional to the time required to duplicate the genomic region (A)–(B). Some of these molecules (marked *rg*) are stained in red and green also within the region (a)–(b). The fraction of *rg* molecules is proportional to the time required to duplicate the genomic region (a)–(b). This relationship is expressed by the equation reported at the bottom of the figure and allows us to calculate *Td_ab_* using parameters that can be easily measured on individual DNA molecules (*N_R_,* the number of *R* molecules; *N_RG_,* the number of *RG* molecules; *N_rg_,* the number of *rg* molecules). Finally, the ratio between the size of the genomic segments analyzed *(L_ab_)* and *Td_ab_* represents the duplication speed of the segment *(Sd_ab_).* The results obtained from the PacI and the SwaI experiments are reported in (B) and (C). Double-headed arrows indicate the genomic segments analyzed quantitatively. Segments marked with the same letter in the two maps correspond to identical portions of the Raji EBV genome. Above each arrow is indicated the corresponding *Sd* value in kilobases per minute. The sizes of these segments are as follows: *A–G,* 25 kb; *H,* 20 kb; *I,* 35 kb; *K,* 10 kb; *L,* 75 kb; and *J,* 10 kb. A comparison of the values obtained from the two experiments shows remarkable similarities. The largest variation was found for segment *G*. However, in both experiments this segment was located at the end of the DNA molecules. Therefore, the variability in stretching in this portion of the molecules may have affected the collection of the data. The red dashed line below the map indicates the position of the RRF.

In the previous sections we showed that different portions of the EBV genome are not equivalent with respect to when and where DNA replication begins and how DNA replication progresses. Here we wanted to determine the quantitative aspects of DNA replication in different portions of the EBV genome. The data obtained by SMARD can be analyzed quantitatively and used to determine the average time required to duplicate any portion of the EBV genome (*Td;* Figure 5A). By knowing *Td* and the length of the segment analyzed, the corresponding duplication speed *(Sd)* can also be calculated (Figure 5A). Importantly, these measurements are based on all the images collected during each SMARD experiment, including the molecules entirely stained in red or in green (several hundred). Therefore, the conclusions reached by this analysis are not solely dependent on the appearance of the immunostaining patterns in a small fraction of the DNA molecules. In addition, the quantitative analysis is performed on relatively large genomic segments; therefore, it is not significantly affected by the resolution at which the positions of the replication forks are determined.

We calculated the value of *Sd* for each portion of the Raji EBV genome, depicted with double-headed arrows in Figure 5 (segments *A–K; Td* is reported in Table 1). The results obtained from the PacI and the SwaI experiments were analyzed independently but show remarkable similarities (compare the values reported above segments marked with the same letter in Figures 5B and 5C). Therefore, the quantitative analysis is highly reproducible.

**Table 1:**
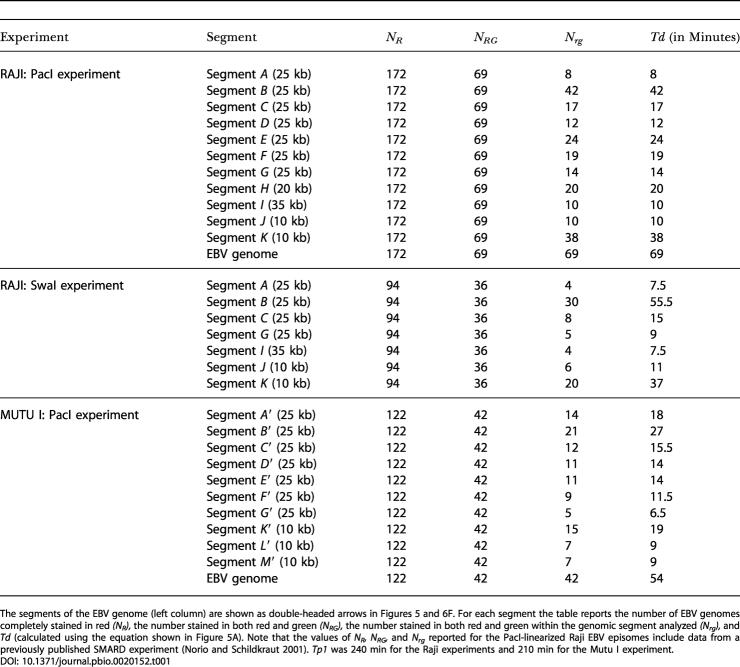
Quantitative Analysis of Different SMARD Experiments

**Figure 6:**
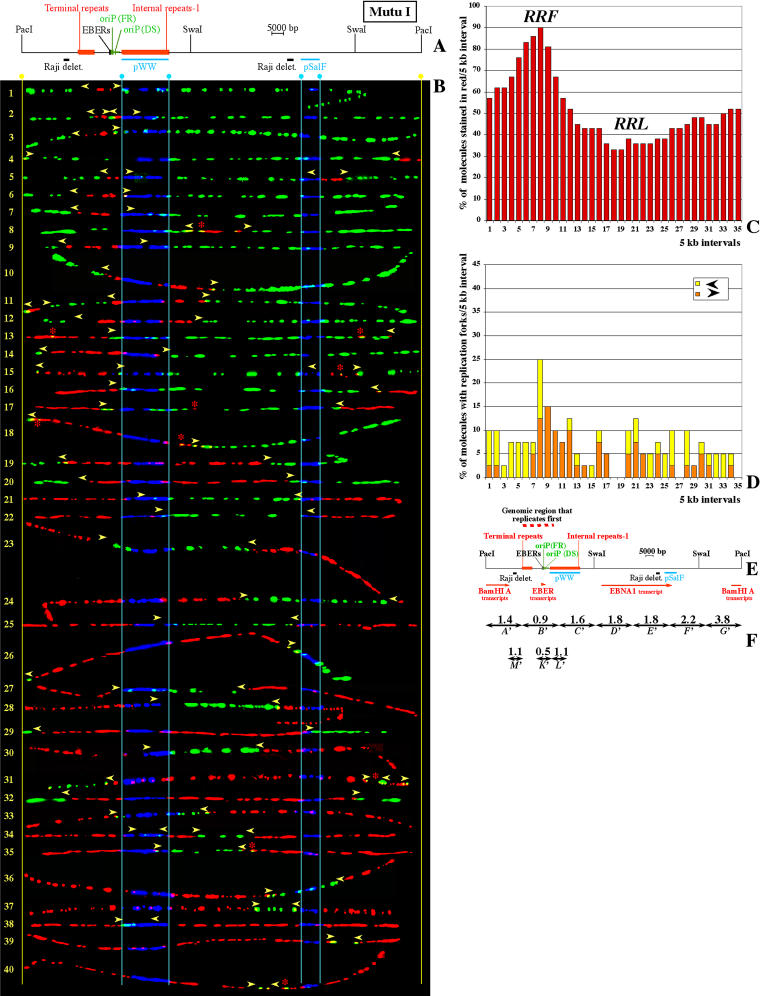
SMARD Performed on PacI-Linearized EBV Episomes Replicated in Mutu I Cells (A) Map of the PacI-linearized EBV genome with the positions of various genetic elements shown to scale. Below the EBV map, light blue bars show the positions of the hybridization probes (pWW and pSalF) utilized to identify the EBV molecules and their orientation. Black bars indicate the positions of two short deletions present in the Raji EBV genome (Polack et al. 1984). (B) Images of 40 PacI-linearized EBV episomes ordered and marked as in Figure 3B (from a population of 42 molecules collected in this experiment). Some molecules are distorted but the positions of the red-to-green transitions are clear. Two additional molecules were unsuitable for precise measurements and are not shown. (C) Replication profile of the PacI-linearized EBV episomes shown in (B). Starting from the PacI site, intervals of 5 kb are indicated on the horizontal axis by numbers from 1 to 35. The vertical axis indicates the percentage of molecules stained red within each 5-kb interval. (D) Profile of replication fork abundance and direction. Intervals of 5 kb are indicated on the horizontal axis as for (C). The vertical axis indicates the percentage of molecules containing replication forks in each 5-kb interval. Three different pausing sites contribute to the significant accumulation of replication forks within interval 8 (the two EBER genes and the FR element). A fourth pausing site (the DS element) is located within interval 9, producing a minor accumulation of replication forks. (E) Map of the EBV genome (to scale) aligned with the horizontal axes of histograms (C) and (D). Red arrows mark the positions of regions transcribed during the *type I* latency that characterize the Mutu I EBV episomes. The red dashed line above the map indicates the position of the RRF. (F) Duplication speed for various segments of the Mutu I EBV genome. Double-headed arrows indicate the genomic segments analyzed quantitatively. Above each arrow is indicated the corresponding *Sd* value in kilobases per minute. Segments *A′–G′* divide the whole EBV genome into seven parts of identical size, corresponding, respectively, to the intervals 1–5, 6–10, 11–15, 16–20, 21–25, 26–30, and 31–35 on the horizontal axes of Figures 3C and 4C. The sizes of these segments are as follows: *A′–G′,* 25 kb; and *K′*, *L′,* and *M′,* 10 kb. Due to the presence of small differences in the DNA sequence (see text), segments *A′–G′* are similar but not identical to segments *A–G* in Figure 5.

The segments of the EBV genome (left column) are shown as double-headed arrows in Figures 5 and 6F. For each segment the table reports the number of EBV genomes completely stained in red *(N_R_),* the number stained in both red and green *(N_RG_),* the number stained in both red and green within the genomic segment analyzed *(N_rg_),* and *Td* (calculated using the equation shown in Figure 5A). Note that the values of *N_R_,* *N_RG_,* and *N_rg_* reported for the PacI-linearized Raji EBV episomes include data from a previously published SMARD experiment (Norio and Schildkraut 2001). *Tp1* was 240 min for the Raji experiments and 210 min for the Mutu I experiment

We found that different portions of the EBV genome replicate at different speeds, with values that range from a minimum of 0.3 kb/min (segment *K;* Figures 5B and 5C) to a maximum of 3.5–4.7 kb/min (segment *I*). More details are provided later in the text. However, it is important to note that the highest *Sd* values were detected within the central portion of the Raji RRF (segments *A, I,* and *G*). This result can be explained in two ways. First, replication forks may move faster throughout the RRF. Alternatively, the RRF could contain a significant level of multiple initiation events. For the reasons mentioned below we favor the second possibility.

### Multiple Initiation Events Can Take Place on the Same Raji EBV Episome within the RRF

Three lines of evidence indicate that multiple initiation events take place within the Raji RRF. Two lines of evidence are discussed in this section (the presence of multiple red patches in the immunostaining patterns of some EBV episomes and the detection of termination events by 2D gel analysis); the third is discussed in the last section of Results (differences in duplication speed across segments of the RRF of different sizes).

The first line of evidence is provided by the immunostaining pattern of the EBV molecules. Although discontinuities in the immunostaining make it difficult to detect multiple initiation events when the distance between converging forks is 5 kb or less, the replication patterns of some of the molecules are compatible with the presence of multiple initiation events within RRF (molecule 8 in Figure 3B and molecules 5 and 9 in Figure 4B). For example, in molecule 9 three regions stained in red (divergent arrows) are separated by two regions stained in green about 4 kb in size (convergent arrows); shorter patches are also visible (asterisks) and might indicate the presence of additional initiation events. In these molecules, the genomic regions stained in red are very close to each other and approach the resolution limits of SMARD. If these signals were produced by multiple initiation events, we should conclude that they took place at about the same time and with a short interorigin distance. Alternatively, secondary initiation events might have taken place in proximity to an incoming replication fork (generated by a primary initiation event). In both cases, the different replication bubbles would rapidly fuse into a larger bubble, making the detection of these events extremely difficult. Since these patterns are too short to be unequivocally ascribed to DNA replication, the presence of multiple initiation events within the RRF was confirmed using a replication mapping approach independent of SMARD.

A second line of evidence is provided by the structure of the replication intermediates examined by 2D gel electrophoresis in exponentially growing Raji cells (see Materials and Methods). We analyzed nine restriction fragments, indicated in Figure 3A as gray bars *(a–i).* We also reexamined the hybridization patterns of ten different fragments analyzed in a previous study (black bars 1–10 in Figure 3A) (Little and Schildkraut 1995). In total, we considered 19 restriction fragments spanning approximately 65% of the Raji EBV genome. The summary of these 2D gel analyses is shown in Figure 3E. Replication intermediates indicative of initiation events were found in several restriction fragments (marked by a green *I* above the corresponding fragment in Figure 3E). The restriction fragments containing replication bubbles are contiguous and span the genomic regions underlined by the red dashed line at the bottom of Figure 3E (approximately corresponding to the RRF). As expected, termination events (marked by a red *T* in Figure 3E) were detected in many of the fragments located within the RRL. Importantly, random termination events were also detected in most of the fragments in which we detected bubble arcs. However, the source of these events could not be identified in our previous studies. As discussed earlier, SMARD shows unequivocally that the RRF completes its duplication before forks originating elsewhere have the time to reach its central portion. We conclude that the termination events detected by 2D gel electrophoresis derive from the collision of replication forks generated by multiple initiation events taking place within the RRF. An estimate of the frequency of these multiple initiation events is reported later in the text.

### In Mutu I Cells, the Order of Activation of the Initiation Sites Varies Throughout the Viral Genome

Previous observations have suggested that some initiation sites (such as oriP) are used at a different frequency in different EBV strains (Little and Schildkraut 1995). However, it was not known whether these changes were the result of modifications in the activity of individual initiation sites or involved multiple initiation sites throughout the EBV genome. In order to determine the extent of these differences, we performed SMARD in another Burkitt's lymphoma cell line called Mutu I (Gregory et al. 1990). A brief description of this cell line and of the conditions used for SMARD is reported in Materials and Methods. From this experiment we recovered the images of 271 PacI-linearized EBV episomes substituted along their entire length with halogenated nucleotides (122 red, 107 green, 42 red and green). The results of this analysis are shown in Figure 6.

The most striking differences in the replication of the two EBV strains were found in the order of activation of the initiation sites. In Raji episomes, primary initiation events are restricted to an 80-kb region approximately corresponding to the RRF (this study and also Norio and Schildkraut 2001). In contrast, in Mutu I episomes primary initiation events occur at many locations throughout the viral genome (molecules 1, 4, 5, and 8; Figure 6B). Multiple initiation events (mostly largely spaced), firing either synchronously (molecules 2 and 5) or asynchronously (molecules 11, 15, 31 and 34), are also frequent in Mutu I. This explains the heterogeneity detected in the positions of the termination events (green patches in molecules 32–40), as well as in the replication patterns of the episomes at intermediate stages of duplication. Hence, DNA replication initiates, progresses, and terminates differently in different Mutu I episomes. Even if the order of activation of the initiation sites varies from molecule to molecule, more than 80% of the Mutu I episomes are stained in red between intervals 6 and 9 (a 20-kb region that includes oriP; Figure 6C). This indicates that this EBV strain also contains an RRF, although its genomic location differs from that found in Raji (compare Figure 3C with Figure 6C). In summary, these results confirm that initiation sites are not confined to a specific portion of the EBV genome and allow us to conclude that their utilization in different viral strains can change throughout the EBV genome.

### The RRFs Are Produced by Clusters of Initiation Sites Frequently Activated at the Beginning of the Duplication of the Episomes

Modifications in the activity of individual initiation sites (such as oriP) could potentially explain the different location of the RRF in Raji and Mutu I episomes. However, the following considerations indicate that this is not the case. Initiation events occurring at oriP take place in the vicinity of the dyad symmetry (DS) element and produce replication forks that stall in the leftward orientation at the family of repeats (FR) element (Gahn and Schildkraut 1989). Initiation events taking place to the left of oriP (such as in Raji episomes) instead produce replication forks that pause in the rightward orientation (interval 8 in Figure 3D). In the Mutu I genome, oriP is at the center of the RRF, but replication forks accumulate in both orientations within interval 8 (see Figure 6D). This indicates that primary initiation events occur on both sides of oriP. Examples of initiation events that took place near the DS element, or to the left of oriP, are visible, respectively, in molecule 5 and in molecules 1 and 3 (Figure 6B). The presence of initiation events on both sides of oriP is also supported by the replication pattern of molecule 2, in which two initiation events spaced approximately 10 kb apart are visible on the same episome. Therefore, in the Mutu I EBV genome, the RRF (∼20 kb in size) contains multiple active initiation sites that have a shared tendency to be activated at the beginning of the duplication of each episome.

Similar conclusions apply to the RRF of the Raji EBV genome (∼80 kb in size), in which primary initiation events were detected at various locations (this study and also Norio and Schildkraut 2001). This could explain why only weak bubble arcs were detected by 2D gel electrophoresis throughout the RRF, even though the duplication of the episomes usually begins within this genomic region (this study and also Little and Schildkraut 1995). We conclude that the RRFs in the Raji and Mutu I EBV genomes are similar in that they contain various initiation sites that have a shared tendency to be activated at the beginning of the duplication of each episome.

### The Duplication Speed of Various Portions of the EBV Genome Is Different in Raji and Mutu I EBV Episomes

In the previous sections we have shown that the order of activation of the initiation sites in Raji and Mutu I EBV episomes is not conserved. Here, we wanted to determine whether the quantitative aspects of DNA replication were also different. SMARD was used to calculate *Sd* for each portion of the Mutu I EBV genome, depicted as double-headed arrows in Figure 6F (segments *A′*–*M′;* see also Table 1). We found that DNA replication proceeds at different speeds throughout different portions of the Mutu I EBV genome (from a minimum of 0.5 kb/min in segment *K′,* to a maximum of 3.8 kb/min in segment *G′*). This is very similar to the range of speeds found in the Raji episomes (0.3–4.7 kb/min). Therefore, DNA replication appears to progress with similar kinetics in the two EBV strains.

We also noticed that similar portions of the EBV genome have different *Sd* values in the two viral strains. Segments *A′–G′* divide the Mutu I EBV genome in seven parts of identical size (∼25 kb; Figure 6F). These segments encompass portions of the EBV genome similar to segments *A–G* in the Raji genome (see Figure 5). However, the values of *Sd* differ significantly in almost every segment. Interestingly, in Mutu I episomes, the highest *Sd* values were not detected within the RRF (segment *G′*). Instead, the RRF contained some of the lowest *Sd* values (segment *B′*). This is probably due to the presence of strong pausing sites within this portion of the Mutu I EBV genome. Nevertheless, segment *B′* replicates faster than the corresponding portion of the Raji EBV genome (segment *B* in Figure 5), a phenomenon that could be explained by the presence of multiple initiation events on both sides of oriP in a fraction of the EBV episomes. In any case, these results indicate that there is no simple correlation between the *Sd* of a genomic segment and its location within the RRF or the RRL.

### Replication Forks Progress at Similar Rates Across Different Portions of the EBV Genome and in Different EBV Strains

Previous observations have suggested that in mammalian cells the speed of the replication forks can vary (Housman and Huberman 1975). Here, we wanted to determine if some of the differences detected in the replication of Raji and Mutu I episomes could be ascribed to modifications in the rate of progression of the replication forks as proposed for other systems (Anglana et al. 2003). For a genomic segment replicated by forks moving in a single direction, *Sd* corresponds to the average speed of the replication forks (provided pausing sites are absent). This allowed us to measure the average speed of the replication forks in various portions of the EBV genomes in which these conditions are satisfied.

In Raji episomes, we found that the average speed of the replication forks was about 1.0 kb/min throughout both segment *H* and segment *J* (see Figure 5); these segments are replicated by forks moving predominantly in one direction (corresponding, respectively, to intervals 22–25 and 9–10 in Figure 3D). Interestingly, a similar value (1.1 kb/min) was found for two different portions of the Mutu I EBV genome in which replication forks also move predominantly in one direction (segments *M′* and *L′* in Figure 6F, corresponding, respectively, to intervals 3–6 and 9–11 in Figure 6D). Therefore, in both EBV strains, we found that the average speed of the replication forks is approximately 1.0 kb/min within every segment that could be analyzed.

Studies performed in different systems have suggested that transcription could interfere with the progression of the replication forks (Brewer 1988; Liu and Alberts 1995). In the Raji EBV genome, segments *J* and *H* (see Figure 5) are located within the long transcription unit of the EBNA genes (see Figure 3F). Throughout segment *J,* replication forks progress in the same direction of transcription, whereas in segment *H* their orientation is reversed (see Figure 3D). Nevertheless, as demonstrated above, replication forks move at the same speed in both segments. Replication forks also move at a similar speed across two nontranscribed regions in the Mutu I EBV genome (segments *M′* and *L′;* see Figure 6). We conclude that in our systems the progression of the replication forks is not significantly influenced by transcription. This could be so either because the level of transcription is not sufficiently high or because, as suggested by others, transcription and DNA replication do not occur at the same time in mammalian cells (Wei et al. 1998).

### The Duplication Speed of a Genomic Segment Reflects the Average Number of Replication Forks Involved in Its Replication

Variation in the utilization of the initiation sites and similarity in the speed of the replication forks suggest that the former should have a stronger influence on the duplication speed of a genomic segment. If we assume that the speed of the replication forks is constant throughout the EBV genome (except for the regions containing pausing sites), *Sd* becomes a function of the number of replication forks actively synthesizing DNA. High *Sd* values would indicate that a large number of replication forks participate in the replication of a genomic segment. If we apply this assumption to the central portion of the Raji RRF, we can see that the values of *Sd* for segments *A* and *G* (see Figure 5) are compatible with the presence 2–3 active forks/segment (corresponding to about one initiation event per duplication cycle within each of these 25-kb segments). Importantly, a larger segment spanning the same portion of the EBV genome (segment *I;* 35 kb in size) replicates even faster (3.5–4.7 kb/min; see Figure 5). This increase could not be explained if the changes in duplication speed were caused by modifications in the speed of the replication forks. However, it is precisely what would be expected if an average of two initiation events take place within the 35-kb segment of the RRF (as suggested by the immunostaining pattern of the episomes and supported by the 2D gel analysis). Therefore, the observed duplication speeds support a model in which, within the RRF, initiation sites spaced 25 kb apart or less can become licensed on the same EBV episome. We also noticed that the EBV genome duplicates faster in Mutu I than in Raji cells (Table 1). This is in agreement with the higher level of widely spaced multiple initiation events detected in Mutu I (compare Figures 3B and 6B). We conclude that the differences in *Sd* across the EBV genome and between the two EBV strains reflect different frequencies of initiation and termination events.

## Discussion

### Conserved and Nonconserved Features in the Latent Replication of Different EBV Strains

In this study, we determined how DNA replication initiates and progresses in EBV episomes latently replicating in two human Burkitt's lymphoma cell lines (Raji and Mutu I). Previous experiments had suggested that some variability in the utilization of oriP might exist among different EBV strains (Little and Schildkraut 1995). Here, however, we found that the replication dynamics vary across the entire EBV genome to an extent that could have not been predicted from previous studies. As exemplified by the replication profiles, the immunostaining patterns of the episomes is strikingly different in the Raji and Mutu I strains (compare Figures 3 and 6). This indicates that the order of activation of the initiation sites is not conserved. Differences were also found in the direction of movement of the replication forks (see Figures 3D and 6D) and in the duplication speed of different portions of the EBV genome (see Figures 5 and 6F). The last, in particular, indicates that the frequency of initiation and termination events varies across the EBV genome and between the two viral strains. We did not find a simple correlation between the *Sd* of a genomic segment and its location within the RRFs. For example, the high *Sd* value for segment *G′* indicates the presence of active initiation sites outside the Mutu I RRF. Therefore the factors that influence the order of activation of the initiation sites are at least partially distinct from the factors that control their frequency of activation.

The EBV episomes replicating in these two cell lines have a similar size and genomic organization. However, the number of internal repeats 1 (also called Bam HI W) is reduced by one unit in the Mutu I strain (not shown), while the Raji EBV genome contains two short deletions (see Figure 6A) (Polack et al. 1984). In principle, these differences could affect some initiation sites. On the other hand, initiation events were detected throughout the EBV genomes. It is unlikely that localized modifications of the DNA sequence (affecting one or few initiation sites) could account for all the differences in the replication of Raji and Mutu I episomes. Primary or secondary events were detected within almost every 25-kb segment of the Mutu I EBV genome (see Figure 6F), such as segment *A′* (molecule 11; see Figure 6B), segment *B′* (molecules 1, 2, 3, and 5), segment *C′* (molecule 34), segment *D′* (molecule 8), segment *F′* (molecules 5 and 15), and segment *G′* (molecules 4 and 31). Similarly, in Raji episomes, replication bubbles were detected within every restriction fragment analyzed by 2D gel electrophoresis throughout a region of about 80 kb (the sizes of these fragments ranged from 3 to 12 kb; Little and Schildkraut [1995] and this study). Using SMARD, low frequencies of secondary initiation events were also detected in the remaining portion of the Raji EBV genome. Therefore, even if SMARD and the 2D gel analysis do not have the resolution to pinpoint the locations of the initiation events at the nucleotide level, our results clearly indicate that the average distance between the initiation sites is below 25 kb.

This study also revealed modifications in the pausing of the replication forks in the oriP region. Accumulation of replication forks is clearly present in both EBV strains within this genomic region. However, only 25% of replicating Mutu I episomes contain replication forks at this location (see Figure 6D), compared with 43% of Raji episomes (see Figure 3D). Quantitative estimates of the average pausing of the replication forks suggested values of about 30 min in Raji episomes and 10 min in Mutu I episomes (data not shown). The decreased pausing could reflect the presence of active initiation sites on both sides of oriP in the Mutu I strain (as suggested by the immunostaining pattern of the episomes). Alternatively, a decreased efficiency of the pausing sites could also contribute to the significant reduction in pausing detected in Mutu I.

We also found that some features of the episomal duplication do not vary. In both Raji and Mutu I cells, replication forks move freely throughout the EBV genome (except near oriP; see Figures 3D and 6D), and their progression rate appears to be constant. This indicates that modifications in the speed of the replication forks do not contribute significantly to the differences in DNA replication described above. This contrasts with results obtained by another laboratory for an amplified genomic locus (Anglana et al. 2003), in which the slower progression of the replication forks—caused by a reduction in nucleotide pools—was presented as the cause for a more frequent activation of initiation sites. Instead, the changes in DNA replication detected in our experiments appear to be caused by differences in the order and frequency of activation of groups of initiation sites encompassing large genomic regions (see next section).

Another common feature between Raji and Mutu I cells is the presence of a genomic region that usually replicates first during the duplication of each episome. The position of this RRF differs in the two EBV strains. However, the direction of movement of the replication forks throughout the RRFs is similar. For example, within the central portion of each RRF, replication forks move in both directions, while along its distal portions, replication forks move predominantly outward (see Figures 3D, 4D, and 6D). We conclude that the direction of movement of the replication forks throughout the EBV genome is mainly a consequence of the dynamics of the initiation of DNA replication.

### Utilization of Initiation Sites is Regulated at the Level of Genomic Regions Rather Than at the Level of Individual Initiation Sites

Even if the EBV episomes utilize the same replication machinery (provided by the host cell), several aspects of their duplication are not conserved between Raji and Mutu I. In mammalian cells, prereplication complexes are believed to form at the end of mitosis, when general transcription is shut off (Okuno et al. 2001; Dimitrova et al. 2002; Mendez and Stillman 2002). However, the selection of specific initiation sites occurs only later in G1, at the origin decision point (Wu and Gilbert 1996). This suggests that there must be a mechanism that, during G1, restricts the utilization of initiation sites to specific regions of the mammalian genomes. In this study, we have shown that initiation sites are present throughout the EBV genome and that their utilization differs dramatically in different EBV strains. It is therefore reasonable to assume that the utilization of the initiation sites in the EBV episomes is restricted by the same mechanisms responsible for the selection of the initiation sites in mammalian chromosomes.

One of the questions we tried to answer is whether initiation of DNA replication is regulated at the level of individual initiation sites. Clues to a possible regulatory mechanism can be found in the replication profiles of the EBV episomes. The RRFs are localized in specific portions of the EBV genome that differ in the two EBV strains. These regions are tens of kilobases in size (about 80 kb in Raji episomes and 20 kb in Mutu I episomes) and encompass multiple initiation sites. The early activation of an individual initiation site could be sufficient to generate a RRF. However, our results have demonstrated that within each RRF various initiation sites have a similar tendency to be activated at the beginning of the duplication of each episome. Therefore, the order of activation of the initiation sites varies at the level of genomic regions rather than at the level of individual initiation sites and might reflect the presence of a particular chromatin organization.

Recent findings have shown that histone acetylation can influence the timing of replication origin firing in yeast (Pasero et al. 2002; Vogelauer et al. 2002). In this study we found that even if initiation events were detected at many locations within the MutuI episomes, primary initiation events occurred predominantly within the RRFs. Modifications in chromatin structure could be used in mammalian cells to regulate the order of activation of the initiation sites across genomic regions that encompass multiple initiation sites. The early activation of the initiation sites located in these regions would increase the chance of passively replicating the neighboring initiation sites contributing, at least in part, to the process of selection of the initiation sites.

In addition to changes in the order of activation of the initiation sites, other mechanisms could influence their utilization by affecting their frequency of activation. We noticed that in Raji episomes the frequency of initiation events across the RRL appears to be reduced compared to that of the RRF. This difference is reflected in the higher levels of *Sd* detected within the RRF compared to the RRL (Figure 5) and in the absence of bubble arcs outside the RRF (Figure 3E). The replication profile of the EBV episomes also indicates that the RRL (the genomic region stained in red in less than 40% of the EBV episomes) is larger in Raji (intervals 9–22 in Figure 3C) than in Mutu I (intervals 17–25 in Figure 6C) and that it mirrors the positions of the longest transcription units active in each strain (see Figures 3E and 6E). This suggests that the presence of a long transcription unit could delay the duplication of the corresponding genomic region. This delay is unlikely to be caused by an impaired progression of the replications forks, since we have shown that the average speed of the replication forks is not significantly influenced by transcription (see Figures 5B, 5C, and 6F). An alternative possibility could be that transcription decreases the frequency of initiation events across the genomic regions traversed by RNA polymerases, as previously suggested by others (Kalejta et al. 1998; Snyder et al. 1988; Haase et al. 1994; Tanaka et al. 1994). Perhaps the passage of RNA polymerase removes, or inactivates, prereplication complexes deposited on the DNA at the end of mitosis (see next section). The observation that initiation events appear more diffusely across the Mutu I EBV genome than in Raji is consistent with this hypothesis and might reflect the presence of larger nontranscribed regions in Mutu I (Gregory et al. 1990). Further experiments will be required to shed light on this phenomenon. However, the detection of some initiation events within the transcribed regions (i.e., molecules 17 and 43 in Figure 3B and molecule 8 in Figure 6B) suggests that the relationship between transcription and replication could be more complex.

### Initiation Sites Are Redundant Elements of the EBV Genome

In this study, we have shown that initiation events are not confined to a specific portion of the episomes, suggesting that DNA sequences capable of functioning as initiation sites must be rather common. This can explain why, under various experimental conditions, individual initiation sites do not appear to play an essential role in the replication of EBV episomes. For example, a hundred-nucleotide deletion encompassing the DS element of oriP is sufficient to abrogate both initiation of DNA replication (Norio et al. 2000) and the binding of ORC and MCM proteins at this genomic location (Chaudhuri et al. 2001; Schepers et al. 2001). This deletion, however, has no apparent effect on the stable replication of the episomes in established cell lines (Norio et al. 2000; Kanda et al. 2001). Therefore, other efficiently licensed initiation sites are present in different portions of the EBV genome.

**Figure 7:**
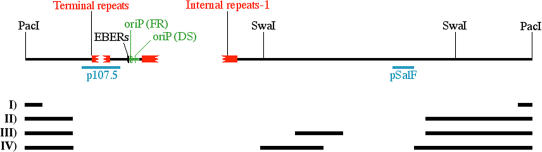
EBV Strains Carrying Large Deletions Map of a generic EBV genome linearized with the restriction endonuclease PacI. The number of terminal and internal repeats 1 can vary in different EBV strains; therefore, broken lines were inserted in the map at these positions. The deletions present in four EBV strains are shown as black bars below the map. Deletion I is 12 kb in size and is present in episomes of cell lines obtained by transformation with B95–8 EBV isolates (Raab-Traub et al. 1980; Parker et al. 1990). This deletion encompasses a portion of the EBV genome corresponding to the central portion of the Raji RRF. The remaining deletions were artificially engineered in the EBV genome. Deletion II is described in Robertson et al. (1994). Deletion III is described in Robertson and Kieff (1995). Deletion IV is described in both Kempkes et al. (1995a) and Kempkes et al. (1995b).

Large deletions are also well tolerated (deletions I, II, III, and IV in Figure 7), even when they encompass portions of the EBV genome known to contain multiple initiation sites (such as the Raji RRF). One of the deleted EBV genomes shown in Figure 7 (genome IV; Kempkes et al. 1995a; Kempkes et al. 1995b) was recently analyzed to detect binding sites for ORC and MCM proteins. Significant binding of these proteins was detected only at oriP (Schepers et al. 2001). However, we found that in this mini-EBV genome, oriP is used at a frequency that approaches 100% (B. Chaudhuri and C. L. Schildkraut, unpublished data). Therefore, the absence/reduction of replication complexes at other locations correlates with an increased usage of the licensed initiation site oriP. Interestingly, these short versions of the EBV genome were specifically engineered to preserve the latency genes by removing most of the untranscribed regions. Therefore, a possible effect of transcription could be to reduce the number of replication complexes present throughout the EBV genome. Reductions in initiation events throughout transcribed regions could be relevant in the maintenance of genomic stability. In fact, it has been reported that at least three extremely large transcription units (*FHIT, WWOX,* and *Parkin;* each ∼1 Mb in size) encompass known common fragile sites in mammalian genomes (Ohta et al. 1996; Ried et al. 2000; Krummel et al. 2002; Denison et al. 2003). We conclude that initiation sites are redundant elements of the EBV genome and that the deletion of some of them can be compensated for by an increased usage of the remaining sites.

### Role of oriP in the Replication of EBV Episomes

Initiation site oriP is the best characterized initiation site of the EBV genome. Initiation of DNA replication has been detected at this site in every EBV strain analyzed to date by 2D gel electrophoresis. However, the frequency of the initiation events at oriP varies in different EBV strains, and it is particularly low in Raji (Little and Schildkraut 1995). The infrequent use of this initiation site in Raji does not appear to be caused by an inability to assemble a prereplication complex. In fact, in this cell line, both ORC and MCM proteins efficiently bind oriP (Chaudhuri et al. 2001; Ritzi et al. 2003). Changes in the DNA sequence of oriP are also an unlikely cause for this difference since only single nucleotide polymorphisms between the Raji and Mutu I strains have been described at this location (Salamon et al. 2000).

In our current study we show that primary initiation events are frequently detected by SMARD near oriP in Mutu I but not in Raji episomes. This could be explained, in part, by the decreased frequency of utilization of this site in the Raji strains. However, this is unlikely to be the only reason. Even infrequent primary initiation events occurring at oriP would produce replication forks that pause in the leftward orientation within interval 8 (as seen in Mutu I), but none of the Raji EBV episomes showed forks paused in this orientation (compare Figure 6D with Figure 3D). Therefore, an alternative explanation is that in Raji episomes the activation of oriP is delayed compared to initiation sites located in the RRF. This would cause an increase in the passive replication of this genomic region, explaining the reduced frequency of activation detected by 2D gel electrophoresis. In this context, the residual activity of oriP in Raji could represent secondary initiation events that take place in proximity to replication forks that originated in the RRF and paused near oriP. These events would produce small red patches, such as the one marked by an asterisk in molecule 35 of Figure 3, that are too close to the paused forks to be unequivocally identified by SMARD as separate initiation events. We conclude that oriP is one of the initiation sites preferentially utilized to begin the duplication of the Mutu I episomes, while in Raji only secondary initiation events usually occur at this site.

Various groups have suggested that different cellular proteins could participate in regulating the activity of oriP (Dhar et al. 2001; Shirakata et al. 2001; Deng et al. 2002). However, it is currently not clear why, in Raji episomes, oriP is not among the preferred initiation sites. Interestingly, it has been reported that oriP is more extensively methylated in Raji than in other EBV strains (Salamon et al. 2000). It is therefore tempting to speculate that epigenetic modifications of the DNA template, or modifications of the chromatin structure, could be responsible for the differences in the order of activation detected in these EBV strains. The epigenetic regulation of oriP activity could be particularly important during the establishment of latent replication, since it has been demonstrated that an epigenetic event is required for the establishment of oriP-dependent replication (Leight and Sugden 2001).

### Conclusions—Flexible Utilization of Initiation Sites in Higher Eukaryotes

In this study we have shown that, while the basic features of DNA replication are conserved (i.e., the progression of the replication forks), the activity of the initiation sites (order and frequency of activation) varies significantly in different EBV strains and across different portions of the EBV genome. Importantly, using SMARD we are now beginning to detect similar modifications in the utilization of initiation sites across transcriptionally active chromosomal loci of the mouse genome (data not shown). Additional mechanisms could regulate DNA replication at transcriptionally silent loci, as suggested by the complete absence of initiation events throughout an approximately 450-kb portion of the mouse IgH locus in non–B cell lines (Zhou et al. 2002; data not shown). These results are compatible with the flexible utilization of initiation sites also suggested by other laboratories (Kalejta et al. 1998; Lunyak et al. 2002). It is therefore likely that the large redundancy in initiation site usage and the regulation of initiation site activity at the level of genomic regions represent common features of DNA replication in mammalian cells. In particular, our results suggest that long-range changes in chromatin structure or chromosomal organization could be far more important than local modifications at individual initiation sites in regulating DNA replication. This could represent an efficient way for eukaryotic cells to control the replication of their very large genomes, and could have broad implications for the maintenance of genomic stability. By using SMARD on primary cells, we will soon be able to determine if similar dynamics are also present in nontransformed mammalian cells.

## Methods

### 

#### Cell cultures, EBV strains, and double-labeling of replicating DNA

Raji cells were grown in exponential phase (as described in Little and Schildkraut 1995), keeping the cell density between 3 × 10^5^ and 8 × 10^5^ cells/ml. The experiments presented in this manuscript were performed at approximately 5 × 10^5^ Raji cells/ml, using two labeling periods (240 min each) with 25 μM IdU (first label) and 25 μM CldU (second label). IdU was added directly to the growing culture, followed by low-speed centrifugation of the cells at the end of the first labeling period and resuspension in warm medium containing CldU.

Early passages of the Mutu I cells (clone c179 p44; Gregory et al. 1990) were provided by Alan B. Rickinson and grown for only seven additional passages (keeping the cell density between 4 × 10^5^ and 8 × 10^5^ cells/ml) before the replicating DNA was labeled. The conditions used for growth and labeling were the same as those used for Raji cells, with the exception that the labeling periods were only 210 min each. This Mutu I cell line is characterized by the presence of a small fraction of cells in which EBV replicates lytically, producing molecules linearized at the terminal repeats. However, this did not affect our analysis of the latently replicating episomes because only the DNA molecules that were circular before the digestion with PacI were recovered from the agarose gels and analyzed by SMARD.

Only ten EBV genes (out of about 100) can be expressed during latency (six EBNAs; two latent membrane proteins [LMPs]; two nontranslated Epstein Barr encoded RNAs [EBERs]; reviewed in Kieff 1996). In EBV-associated diseases, where the viral genome is maintained as a circular episome, the phenotype of the infected cell influences the viral patterns of expression (Babcock et al. 2000). Three different latent transcription patterns have been described (Kerr et al. 1992): *type I* (only *EBNA1* and EBERs expressed), type *II* (only *EBNA1,* the LMPs, and the EBERs expressed), and type *III* (all the EBNAs, the LMPs, and the EBERs expressed). Although both Raji and Mutu I are human Burkitt's lymphoma cell lines, their transcription profiles are different. The Mutu I cell line used in this study was an early passage of a *type I* clone isolated in the Alan B. Rickinson laboratory. Raji cells, instead, have a *type III*-like transcription pattern and they also carry a deletion of the *EBNA3C* gene (Polack et al. 1984).

#### Improved method to stretch a large number of EBV molecules on individual slides

In order to collect a sufficient number of images of the EBV genome, the population of replicated episomes needed to be enriched by a partial purification using pulsed field gel electrophoresis. However, starting from the limited amount of DNA that can be purified from a pulsed field gel, we could not stretch a sufficient number of DNA molecules on microscope slides by molecular combing (Bensimon et al. 1994). As a consequence, the collection of several hundred images of EBV episomes would have required the analysis of a very large number of microscope slides and the use of large amounts of pulsed field–purified DNA. In order to solve this problem, we decided to stretch the DNA molecules using a modification of the method originally introduced for the optical mapping of restriction sites on individual DNA molecules (Aston et al. 1999 and references therein) as well as for other applications (Henegariu et al. 2001). The stretching was achieved by the movement of a DNA solution (a few microliters) gently deposited at the interface between a silanized microscope slide and a nonsilanized coverslip. In this way it was possible to complete our analysis using just few microscope slides and a fraction of the pulsed field–purified DNA derived from the digestion of 10^6^ cells. The molecules stretched by capillary action vary in their orientation (see Figure 1) and in their size (see Figure 2A). Nevertheless, the EBV molecules were clearly identified by the two hybridization signals. These images were aligned with the map of the EBV genome by computer adjustment of the image size for the entire DNA molecule (see Figures 2B and 2C), as we did previously when EBV episomes were stretched by molecular combing (Norio and Schildkraut 2001).

#### Hybridization, probe detection, and immunostaining of the individual DNA molecules stretched on microscope slides

Hybridization was performed as previously described (Parra and Windle 1993) using probes prepared by nick translation in the presence of biotin-16-dUTP (Roche, Basel, Switzerland). The probes used in this study, pSalF, p107.5, and pWW (provided by John L. Yates), were detected using a modification of the DIRVISH procedure (Heng et al. 1995). Briefly, five layers of Alexa Fluor 350 conjugated NeutrAvidin (Molecular Probes, Eugene, Oregon, United States) and biotinylated anti-avidin antibodies (Vector Laboratories, Burlingame, California, United States) were deposited on the microscope slide, washing with PBS, 0.03% Igepal CA-630 (Sigma, St. Louis, Missouri, United States) after each step. The purpose of the hybridization signals is to identify and orient the EBV episomes. Since the DNA molecules studied by SMARD are substituted along their entire length with halogenated nucleotides, they are very easy to detect even in presence of substantial hybridization background (i.e., blue dots in the lowest panel of Figure 1). This hybridization background does not affect SMARD, therefore it was digitally removed from the images of the molecules shown in Figures 3B, 4B, and 6B (as described in other studies performed on stretched DNA molecules; Pasero et al. 2002).

Immunostaining to detect IdU and CldU was performed simultaneously with the detection of the biotinylated DNA probes. Mouse anti-IdU (Becton-Dickinson, Palo Alto, California, United States) and rat anti-CldU (Accurate Chemical, Westbury, New York, United States) were used as primary antibodies (monoclonal), while Alexa Fluor 568-conjugated goat anti-mouse (Molecular Probes) and Alexa Fluor 488-conjugated goat anti-rat (Molecular Probes) were used as secondary antibodies. The immunostaining has almost no background. As described previously (Norio and Schildkraut 2001), the specificity of the immunostaining was tested on DNA fully substituted with IdU or with CldU. No cross-reaction of the antibodies was detected using the detection procedure utilized in this study, and both antibodies were unable to recognize the unlabeled DNA. In practice, the background visible in the red and green channels is mainly represented by other DNA molecules containing halogenated nucleotides (white horizontal arrowheads in the central panels of Figure 1). These molecules can be fully or partially stretched (sometimes collapsed or broken in pieces), but are usually clearly distinguishable from the unbroken, fully substituted EBV molecules. By using appropriate dilutions of the DNA sample during stretching, we minimized the overlap of different molecules.

#### Advantages in the labeling scheme utilized for SMARD and internal controls

Studies performed by fiber autoradiography have previously shown that the results obtained using DNA fibers (such as the average size of the replicons) are significantly affected by the length of the labeling period utilized to label the replicating DNA (reviewed in Berezney et al. 2000). In these studies bias could also be introduced during the collection of the data as a result of the criteria utilized by the experimenter in the choice of the images analyzed. In addition, if synchronized cells are considered, the length of the labeling period also defines the potential resolution at which initiation sites can be mapped, and the estimate of the replication fork speed. Replacing the radioactive detection of the labels with fluorescence microscopy does not solve any of these problems, nor does the statistical analysis of the data.

These problems are completely eliminated by the labeling scheme that characterizes SMARD (Norio and Schildkraut 2001). For our experiments we utilized exponentially growing cells and labeling periods that are longer than the time required to fully replicate the genomic region of interest. In practice, since the replication of a specific genomic region can proceed differently in various DNA molecules, we utilize labeling periods that are sufficiently long to insure the duplication of even the slowest replicating molecules. In addition, only the molecules completely replicated during these labeling periods are examined. By studying this particular population of molecules, we introduce an objective criterion in the collection of the data, eliminating possible biases. Therefore, the molecules replicated during these labeling periods will faithfully represent the distribution of the replication forks in the steady-state population of replicating molecules (Norio and Schildkraut 2001).

Using long labeling periods, and limiting our analysis to the molecules entirely substituted with the halogenated nucleotides, also provides multiple internal controls. These controls cannot be performed when short labeling periods are used. Since the molecules are immunostained throughout their length, their images can be easily aligned to the map of the genomic region analyzed. This allows us to detect the presence of unevenly stretched molecules that can therefore be discarded. In addition, since the immunostaining is visible along the entire length of the molecules, the loss of signal caused by the breakage of some molecules is immediately revealed. The complete substitution of the DNA molecule with halogenated nucleotides also allowed us to easily detect overlaps between different DNA molecules. These overlaps can occur relatively frequently during the stretching of the molecules and their frequency increases as the density and the size of the DNA molecules increases. It is also worth noting that the presence of hybridization probes decreases the intensity of the immunostaining along the corresponding portion of the DNA molecules (see Figure 1). This causes a significant loss of information along the hybridized regions, but it also represents an additional control indicating that the immunostaining is indeed present on the DNA molecules that we intend to study (rather than on adventitiously overlapping molecules). Finally, our labeling scheme allows us to insure that the replication proceeded normally during the labeling of the replicated DNA and that no bias was introduced during the collection of the images. In fact, when these conditions are satisfied, the number of molecules fully substituted with IdU is expected to be very similar to the number of molecules fully substituted with CldU. These controls represent a strong proof that the images of the molecules are representative of a steady-state population of replicating molecules.

#### Analysis of the replication intermediates by 2D gel electrophoresis at neutral pH

The procedures for the enrichment of replication intermediates, 2D gel electrophoresis, and Southern analysis were essentially as described previously (Little and Schildkraut 1994; Norio et al. 2000). Preparations of replication intermediates from Raji cells were digested with different restriction enzymes depending on the fragment analyzed: EcoRI/DraI for fragments *a–e,* EcoRI/EcoRV for fragments *f* and *I,* EcoRI/HindIII for fragment *g,* and EcoRI/XbaI for fragment *h*. The positions (EBV strain B95–8 coordinates) of the restriction fragments analyzed by 2D gel electrophoresis were as follows: fragment *a,* DraI (79202)–EcoRI (82920); fragment *b,* EcoRI (82920)–DraI (88865); fragment *c,* DraI (88865)–EcoRI (91421); fragment *d,* EcoRI (93162)–EcoRI (95239); fragment *e,* EcoRI (95239)–DraI (103226); fragment *f,* EcoRV (100583)–EcoRV (116863); fragment *g,* HindIII (110942)–EcoRI (125316); fragment *h,* XbaI (161383)–EcoRI (1); and fragment *I,* EcoRV (126415)–EcoRI (137221). The probes used to detect the restriction fragments were as follows: pHindLHI for fragments *a–c,* pHindE for fragments *d–e,* pSalF for fragment *f,* the pHindC fragment XbaI (121146)–BglII (120341) for fragment *g,* the p107.5 fragment XhoI (169423)–XhoI (167487) for fragment *h,* and the pHindC fragment HpaI (131959)–XbaI (133151) for fragment *i*. The plasmids pHindLHI, pHindE, pSalF, pHindC, and p107.5 were kindly provided by John L. Yates. Two different preparations of replication intermediates were used to study the replication patterns of fragments *a–c.*

## Abbreviations

2D: two-dimensional
CldU: 5′-chloro-2′-deoxyuridine
DS: dyad symmetry
EBER: Epstein Barr encoded RNA
EBNA: Epstein Barr nuclear antigen
EBV: Epstein-Barr virus
FR: family of repeats
IdU: 5′-iodo-2′-deoxyuridine
LMP: latent membrane protein
RRF: region that usually replicates first
RRL: region that usually replicates last
Sd: duplication speed
SMARD: single molecule analysis of replicated DNA
Td: duplication time
